# Identification and characterization of Smyd2: a split SET/MYND domain-containing histone H3 lysine 36-specific methyltransferase that interacts with the Sin3 histone deacetylase complex

**DOI:** 10.1186/1476-4598-5-26

**Published:** 2006-06-28

**Authors:** Mark A Brown, Robert J Sims, Paul D Gottlieb, Philip W Tucker

**Affiliations:** 1 Section of Molecular Genetics and Microbiology and Institute for Cellular and Molecular Biology, University of Texas at Austin, Austin, Texas 78712, USA; 2 Present address: Division of Nucleic Acids Enzymology, Department of Biochemistry, Robert Wood Johnson Medical School, Piscataway, New Jersey 08854, USA

## Abstract

**Background:**

Disrupting the balance of histone lysine methylation alters the expression of genes involved in tumorigenesis including proto-oncogenes and cell cycle regulators. Methylation of lysine residues is commonly catalyzed by a family of proteins that contain the SET domain. Here, we report the identification and characterization of the SET domain-containing protein, Smyd2.

**Results:**

Smyd2 mRNA is most highly expressed in heart and brain tissue, as demonstrated by northern analysis and *in situ *hybridization. Over-expressed Smyd2 localizes to the cytoplasm and the nucleus in 293T cells. Although accumulating evidence suggests that methylation of histone 3, lysine 36 (H3K36) is associated with actively transcribed genes, we show that the SET domain of Smyd2 mediates H3K36 dimethylation and that Smyd2 represses transcription from an SV40-luciferase reporter. Smyd2 associates specifically with the Sin3A histone deacetylase complex, which was recently linked to H3K36 methylation within the coding regions of active genes in yeast. Finally, we report that exogenous expression of Smyd2 suppresses cell proliferation.

**Conclusion:**

We propose that Sin3A-mediated deacetylation within the coding regions of active genes is directly linked to the histone methyltransferase activity of Smyd2. Moreover, Smyd2 appears to restrain cell proliferation, likely through direct modulation of chromatin structure.

## Background

Cell proliferation and differentiation are coordinated by synchronized patterns of gene expression. The regulation of these patterns is achieved, in part, through epigenetic mechanisms that affect the nature of DNA packaging into chromatin [1]. Specifically, post-translational covalent modifications to histone tails impact the structural dynamics of the nucleosome, thereby affecting DNA accessibility to transcriptional complexes [2-4]. Common modifications to histones include methylation, acetylation, phosphorylation, and ubiquitination [5]. Importantly, alterations in global levels of histone methylation and acetylation are connected to the biology of cancerous lesions and their clinical outcome [6]. A number of histone lysine methyltransferases (HKMTs) are disrupted in a variety of cancer types [7,8]. How histone methylation mechanistically contributes to the oncogenic state is poorly understood.

All known HKMTs, with one exception [5], catalyze methyl transfer via the SET domain, a module encoded within many proteins that regulate diverse processes, including those critical for development and proper progression of the cell cycle [2,9,10]. Histone lysine methylation on specific residues typically correlates with distinct states of gene expression [5]. Histone 3 (H3) contains most of the known targeted lysines of histone methyltransferases and thereby serves as a conduit of such epigenetic regulation. In general, lysine methylation on H3K9, H3K27, and H4K20 corresponds with gene silencing, whereas methylation of H3K4, H3K36, or H3K79 is associated with actively transcribed genes [5]. Methylation of H3K36 (H3K36me) is tightly associated with actively transcribed genes [11,12], and appears to correspond primarily within the coding region. H3K36 methylation by Set2 in yeast was recently observed to recruit an Rpd3-mediated histone deacetylase complex through direct recognition of H3K36me by the chromodomain of Eaf3 [13-15]. Rpd3 is a histone deacetylase (HDAC) that has well-established functions as a transcriptional repressor [13]. Rpd3 associates into several co-repressor complexes, including one that contains Pho23, Sds3, Sap30, Ume1, Cti6/Rxt1, and Sin3 [13]. However, recent evidence suggests that HDACs may also play a role during active transcription. As such, methylation of H3K36 is directly linked to histone deacetylation via Rpd3-Sin3 that in turn functions to maintain chromatin structure during active transcription [13-15]. These findings reveal a new level of complexity with respect to histone modifications, and demonstrate our need to better understand the enzymes that catalyze these modifications.

**Figure 1:**
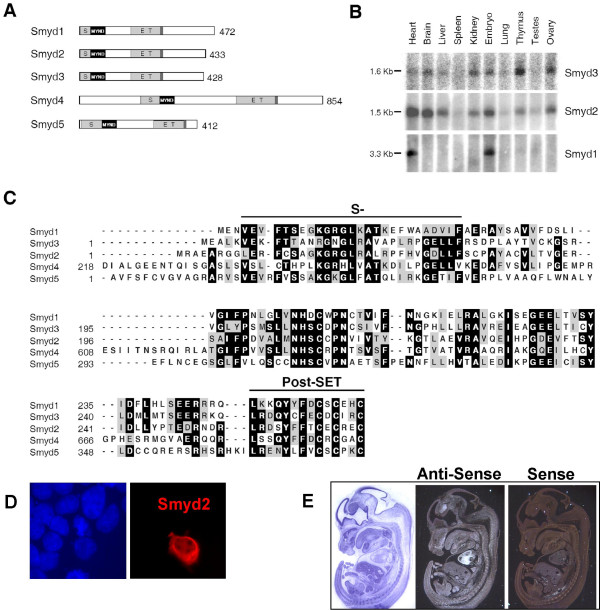
**Alignment of the mammalian Smyd family proteins, and Smyd2 localization. **(A) Schematic representation of the five mammalian Smyd proteins. The split SET domain is shown in light gray; the MYND domain is represented in black and the cysteine-rich post-SET domain is displayed in dark gray. Positions of the amino acids are indicated. (B) Expression of Smyd1, Smyd2, and Smyd3 transcripts in tissues. Top panel: Smyd3 mRNA is most highly expressed in the thymus and in skeletal muscle [8]. Middle panel: Smyd2 mRNA is most highly expressed in the heart and brain. Bottom panel: Smyd1 expression is restricted to the heart and skeletal muscle [20]. Transcripts for Smyd1, Smyd2, and Smyd3 are expressed in the embryo. (C) ClustalW alignment of the amino-terminal SET residues, the MYND domain, and the core SET residues followed by the post-SET domain present in Smyd1, Smyd2, Smyd3, Smyd4, and Smyd5. (D) Smyd2 is localized to the cytoplasm and the nucleus. Exponentially growing 293T cells were transfected with 1 μg of plasmids, encoding myc tagged Smyd2. 48 hr post-transfection, cells were fixed, washed, permeabilized and exposed to monoclonal mouse anti-myc antibody. Nuclei were counterstained with DAPI. Right panel: Smyd2 (red) localizes to both the nucleus and the cytoplasm of 293T cells. Left panel: Nuclei were counterstained with DAPI (blue). The experiments were repeated in triplicate with identical results. (E) Smyd2 mRNA is localized in the heart and hypothalamus of the brain at E13.5. Whole-mount *in situ *hybridization of Smyd2 transcripts in embryos at day 13.5 post coitus were prepared by exposition of sense (right panel) and antisense (middle panel) DNA probes, specific to Smyd2 to the sections. Whereas hybridization with sense probe resulted in no signal (right panel), thus, serving as control, Smyd2 mRNA is easily detected in the heart and the hypothalamus of the brain in embryos at day 13.5 post coitus (middle panel).

Here we describe a subfamily of SET domain containing proteins with a unique domain architecture. This family of proteins is defined by a SET domain that is split into two segments by an MYND domain, followed by a cysteine-rich post SET domain [16] (Fig. 1A). Members of this family may be important developmental regulators, as targeted disruption of the Smyd1 gene results in impaired cardiomyocyte maturation, flawed cardiac morphogenesis, and embryonic lethality [17]. Functionally, Smyd1 is thought to regulate gene expression via its association with histone deacetylase activity [17]. Smyd3 has been noted for its involvement in cancer cell proliferation [8]. It is over-expressed in most hepatocellular and colorectal carcinomas, and its exogenous over-expression in NIH3T3 cells significantly augmented growth [8,18,19]. Similar to Smyd1, Smyd3 modulates chromatin structure through its intrinsic H3K4-specific HKMT activity [8]. Although Smyd2 is highly conserved with Smyd1 and Smyd3, nothing is known about its biochemical or functional activities. Here, we demonstrate that Smyd2 contains SET-domain dependent H3K36 HKMT activity. Smyd2 specifically associates with the Sin3A histone deacetylase complex, suggesting a link between two independent chromatin modification activities. Moreover, we observe that over-expression of Smyd2 in NIH3T3 cells significantly suppresses their growth. We propose that Smyd2-mediated chromatin modification regulates specific gene expression that has important implications for normal and neoplastic cell proliferation.

## Results

### Structural characteristics and expression of Smyd2

Although there are over 50 SET domain-containing proteins encoded in the human genome, only a fraction have been shown to methylate histones. Of all the SET proteins, five cluster into a sub-family that contains a SET domain that is split into two segments by a MYND domain/zinc-finger motif, implicated previously in protein-protein interactions (**S**ET and **MY**N**D**)(Fig. 1C). Members of this family are direct regulators of cancer (Smyd3) and essential developmental processes (Smyd1) [8,17]. Thus, it is important to discern the biochemical and biological properties of Smyd2, given its high degree of homology to Smyd1 and Smyd3. Data from Expressed Sequence Tags suggest that Smyd2 is expressed in a wide range of normal, tumor, and diseased tissues (data not shown). To determine the tissues of highest gene expression, northern blotting was performed with a multiple tissue blot using a probes specific to Smyd1, Smyd2, or Smyd3. The northern analyses (Fig. 1B) demonstrate that in contrast to Smyd1, Smyd2 and Smyd3 mRNAs are expressed more broadly in a wide variety of tissues. Smyd1 is expressed only in T lymphocytes, heart muscle, and skeletal muscle, as previously reported [20]. Smyd3 expression is highest in skeletal muscle [8] and thymus (Fig. 1B), although its transcripts are also highly detected in the brain, kidney, and ovary (Fig. 1B). Tissues containing highest levels of Smyd2 mRNA transcripts include heart, brain, liver, kidney, thymus, and ovary (Fig. 1B.) Additionally, both Smyd2 and Smyd3 transcripts are detectable in embryonic mRNA, suggesting that as with Smyd1, Smyd2 and Smyd3 may be involved in development (Fig. 1B). To determine which embryonic tissues manifest highest levels of Smyd2 transcripts, whole-mount *in situ *hybridization was performed using murine embryos at day 13.5 with a probe specific to Smyd2. At this mid-gestation stage, Smyd2 transcripts are localized to the heart and the hypothalamus of the brain (Fig. 1E).

Immunohistochemical staining of Smyd1 and Smyd3 indicated that both proteins localize within the cytoplasm and nucleus of C2C12 [21] and Huh7 cells [8], respectively. To determine the subcellular localization of Smyd2, immunohistochemical staining was performed using a myc-tagged Smyd2 fusion protein. Similar to Smyd1 and Smyd3, Smyd2 localizes within both the cytoplasm and the nucleus (Fig. 1D).

### Smyd2 is a SET-dependent HKMT

**Figure 2:**
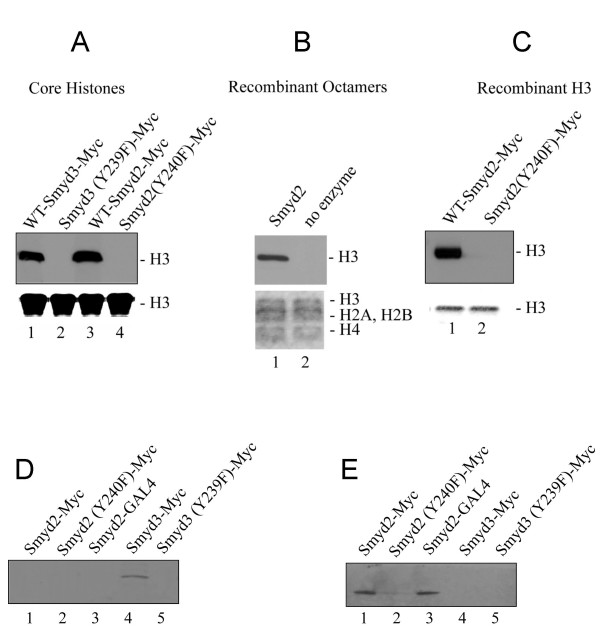
**Smyd2 dimethylates Histone H3 lysine 36**. (A) Smyd2 methylates histone H3 in an *in vitro *histone methyl transferase (HKMT) assay using mixed histones from calf thymus as substrate. Fluorograms are shown in the upper panel; the 17 kD band, corresponding with Histone H3, is indicated; Coomassie stained SDS-PAGE gels were used to verify equal loading and are depicted in the lower panels. Lanes 1 and 3 show positive HKMT activity at H3 by myc tagged Smyd3 and myc tagged Smyd2, respectively. Lanes 2 and 4 indicate that neither the Smyd3 (Y239F) nor the Smyd2 (Y240F) catalytic mutants have HKMT activity. It is concluded that the HKMT activity of Smyd3 depends on Y239 and Y240 for Smyd2. (B) Smyd2 methylates Histone H3 in an *in vitro *histone methyl transferase assay using recombinant octamers as substrate. Fluorograms are shown in the upper panel; the 17 kD band, corresponding with Histone H3, is indicated; Coomassie stained SDS-PAGE gels were used to verify equal loading and are depicted in the lower panels. Histone H3 was found methylated by Smyd2 using recombinant octamers as substrate in an *in-vitro *HKMT assay. (C) Smyd2 methylates histone H3 in an *in vitro *histone methyl transferase assay using recombinant histone H3 as a substrate. Fluorograms are shown in the upper panel; the 17 kD band, corresponding with histone H3, is indicated; Coomassie stained SDS-PAGE gels were used to verify equal loading and are depicted in the lower panels. Histone H3 was found methylated by Smyd2 using recombinant octamers as substrate in an *in-vitro *HKMT assay (lane 1). The catalytically defective mutant Smyd2 (Y240F) failed to methylate recombinant histone H3 (lane 2). It is concluded that the HKMT activity of Smyd2 depends on Y240. (D) Smyd2 does not dimethylate histone H3 at lysine 4 using recombinant histone H3 as a substrate in an *in-vitro *HKMT assay. Western results, using antibodies, specifically reactive with dimethylated histone H3, lysine 4, are shown; the 17 kD band, corresponding with histone H3, is indicated. Lanes 1 and 4 indicate that immunoprecipitated and myc-tagged Smyd3, but not myc-tagged Smyd2, dimethylates histone H3 at lysine 4. Lanes 2 and 5 show that neither Smyd2 (Y240F) nor Smyd3 (Y239F) dimethylate histone H3 at lysine 4. We conclude that Smyd2 does not dimethylate histone H3 at lysine 4. (E) Smyd2 dimethylates histone H3 lysine 36 using recombinant histone H3 as a substrate in an *in-vitro *HKMT assay. Western results, using antibodies, specifically reactive with dimethylated histone H3, lysine 36, are shown; the 17 kD band, corresponding with histone H3, is indicated. Lanes 1 and 3 indicate that Smyd2 dimethylates recombinant histone H3 at lysine 36, independent of the myc or Gal4 tag. The catalytically inactive mutant Y240F does not dimethylate recombinant histone H3 at lysine 36 (lane 2). Smyd3, as well as the catalytically defective mutant Y239F, do not dimethylate recombinant histone H3 at lysine 36 (lanes 4 and 5). We conclude that Smyd2 dimethylates recombinant histone H3 at lysine 36, whereas Smyd3 does not display this activity.

Smyd2 contains the catalytic core residues of the SET domain shown to be critical for the histone methyltransferase activity of Smyd1 and Smyd3 (Fig. 1C) [22,8]. This suggests that Smyd2 may also possess HKMT activity. Histone methylation was tested after incubation of wild-type Smyd2-Myc (or Smyd3-Myc as a positive control) with S-adenosyl-L – [methyl-^3^H ] methionine (SAM) and mixed histones from calf thymus as a substrate. A 17 kD band corresponding to ^3^H-labelled H3 was seen with both Smyd3 and Smyd2 in the fluorogram (Fig. 2A, lanes 1 and 3), indicating that Smyd2 has intrinsic HKMT activity. A tyrosine in the C-terminal region of the core SET domain is conserved among catalytically active SET domain proteins (Fig. 1C). Therefore, to test if the SET domain is required for the HKMT activity observed for Smyd2, point mutations were made in this residue of Smyd3-Myc (Y239F) and Smyd2-Myc (Y240F). Neither Smyd3 (Y239F)-Myc nor Smyd2 (Y240F)-Myc displayed HKMT activity (Fig. 2A, lanes 2 and 4).

Smyd2 HKMT activity was next tested on a recombinant histone H3 substrate. Wild-type Smyd2-Myc methylated H3 (Fig. 2C, lane 1) but Smyd2 (Y240F)-Myc showed no HKMT activity (Fig. 2C, lane 2). To test the activity of untagged Smyd2 protein, Smyd2 was expressed as a GST fusion in E. coli and the GST moiety was subsequently cleaved. Methylation of recombinant histone octamers was tested after incubation with recombinant Smyd2. Consistently, recombinant Smyd2 was observed to methylate histone H3 (Fig. 2B, lane 1). Therefore, we conclude that the Smyd2 has SET domain-dependent, intrinsic HKMT activity.

### Smyd2 dimethylates H3K36

Given that both Smyd1 and Smyd3 have been shown to have specificity for H3K4 [22,8], we tested whether Smyd2 has similar specificity. Histone methyltransferase assays were performed using recombinant H3, and specificity was determined by western blotting using antibodies against various methyl-lysine residues. The antibodies used in these assays include anti-dimethyl H3K4, anti-trimethyl H3K4, anti-dimethyl H3K9, anti-trimethyl H3K9, anti-trimethyl H3K27, anti-di-methyl H3K36, and anti-dimethyl H3K79. A 17 KD band corresponding to H3 was observed with Smyd3, but not with Smyd2, when reactions were probed with anti-dimethyl H3K4 (Fig. 2D, lanes 4 and 1, respectively) or anti-trimethyl H3K4 (data not shown). This indicated that Smyd2 has a different target specificity than Smyd1 or Smyd3. Instead, the HKMT activity of Smyd2 was specific for H3K36, as determined by western blotting with anti-dimethyl H3-K36 antibodies (Fig. 2E, lanes 1 and 3). No additional residues appeared to be targeted by Smyd2 using the other antibodies listed above (data not shown). Therefore, we conclude that Smyd2 dimethylates H3K36.

### Smyd2 associates with HDAC1 and the Sin3 repression complex

**Figure 3:**
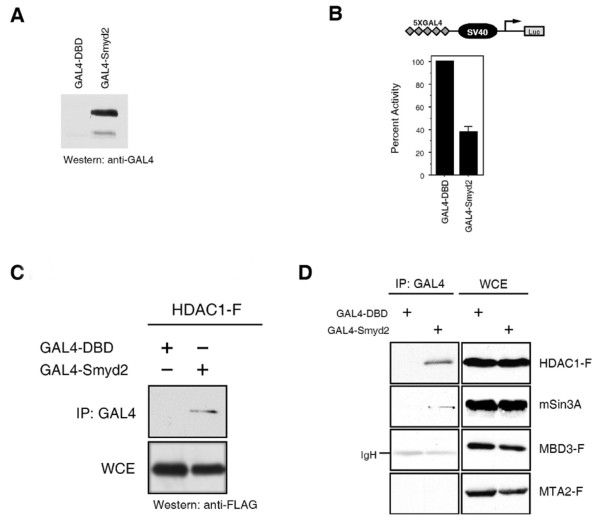
**Smyd2 associates with the Sin3 repression complex and is involved in transcriptional repression. **(A) Expression of GAL4-Smyd2 fusion protein in 293T cells. Exponentially grown 293T cells were transfected with the constructs indicated and, 48 hours post transfection, whole cell lysate was prepared using RIPA buffer and subjected to western analysis using antibodies directed against the GAL4 tag. A reactive band was detected at the appropriate molecular weight (approximately 66 kD). Extracts from cells, transfected with the GAL4-DBD construct [17], served as negative control. (B) Smyd2 represses transcription of a luciferase reporter. Top panel: Schematic illustration of the reporter construct used. Bottom panel: 10T1/2 cells were transiently co-transfected with the 5XGAL4-SV40-luciferase reporter (1 μg) together with GAL4-DBD or GAL4-Smyd2 (2 μg each). Percent activity of the luciferase was determined in relation to GAL4-DBD. Smyd2 significantly represses the transcription of a luciferase reporter in 10T1/2 cells. (C) Smyd2 associates with HDAC1. Exponentially grown 293T cells were transiently co-transfected with GAL4-DBD or GAL4-Smyd2, together with Flag tagged HDAC1 (HDAC1-F). Whole cell RIPA extracts were immunoprecipitated using an anti-GAL4 antibody and immunoblots were probed with an anti-FLAG antibody. As shown here, Smyd2 associates with HDAC1. RIPA whole cell extracts from GAL4-DBD transfected cells [17] served as negative control. Equal protein amounts in the immunopreciptation assays was demonstrated by analysis of 5% input using anti Flag antibodies. (D) Smyd2 interacts with the Sin3A but not the NuRD complex. Exponentially grown 293T cells were transfected with the constructs indicated and, 48 hours post transfection, whole RIPA lysate was prepared. Antibodies directed against GAL4 were used for immunoprecipitation, followed by western analysis using the antibodies indicated. Smyd2 associates with HDAC1 and Sin3A but not with the components of the NuRD complex, MBD3 or MTA2.

Smyd3 induces transcriptional activation by binding to specific promoter sequences [8]. In contrast, Smyd1 is known to repress transcription when fused to GAL4 by association with HDAC activity [17]. Given that Smyd2 has activity towards H3K36, a mark associated with active transcription, we tested the transcriptional regulatory activity of Smyd2. A GAL4-fusion protein was generated using Smyd2 and transient luciferase assays were performed in 10T1/2 cells. Unexpectedly, Smyd2-GAL4 inhibited transcription from an SV40 promoter that contained GAL4 binding sites (Fig. 3B), suggesting it may function similarly to Smyd1.

Although methylation of H3K36 is associated with actively transcribed genes, three recent reports have demonstrated that in yeast, methylation of H3K36 by Set2 recruits an Rpd3-Sin3 histone deacetylase complex [13,14]. Our finding that Smyd2 contains H3K36 methylation activity and functions to repress transcription in the above assay prompted us to investigate whether Smyd2 interacts with the human homologues of the Rpd3-Sin3 complexes. In transient transfection experiments in 293T cells, the human homologue of Rpd3, HDAC1, interacted specifically with Smyd2-GAL4 upon immunoprecipitation with anti-GAL4 antibodies (Fig. 3C). Consistently, when cell extracts from 293T cells over-expressing Smyd2-GAL4 were immunoprecipitated with anti-GAL4 antibody, the immune complexes contained endogenous Sin3A (Fig. 3D). In contrast, Smyd2-GAL4 failed to coimmunoprecipitate FLAG-tagged MBD3 and MTA2, components of the HDAC1-containing NuRD complex (Fig. 3D). Thus, we conclude that Smyd2 preferentially interacts with distinct HDAC1-containing complexes, namely Sin3A.

### Smyd2 suppresses cell proliferation

**Figure 4:**
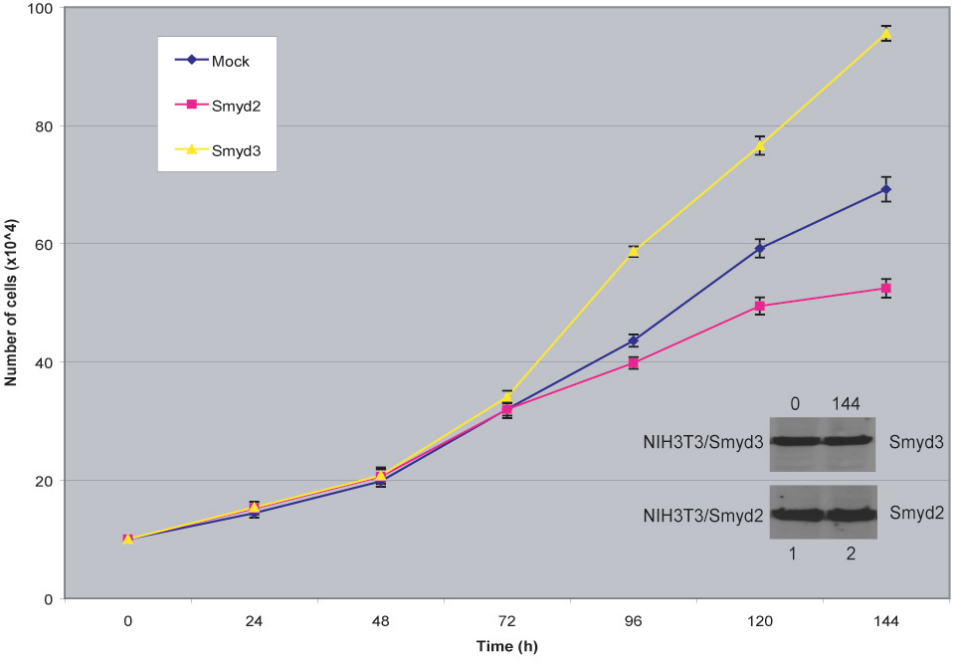
**Smyd2 suppresses NIH3T3 cell proliferation. **Exponentially grown NIH3T3 cells were transfected with plasmids encoding myc-tagged Smyd2 or myc-tagged Smyd3. Cells, transfected with the empty expression construct (Mock), served as control. All cells were monitored by cell counting using trypan blue exclusion. The inserts show the level of expression of Smyd2-myc and Smyd3-myc at 0 and 144 hours post transfection, demonstrating similar levels of ectopically introduced proteins in the NIH3T3 cell lines used. Whereas ectopically introduced Smyd3 enhanced the proliferation, Smyd2 displayed a negative effect on the growth rate of NIH3T3 cells.

The role of Smyd3 in transcriptional regulation as a histone methyltransferase has been linked to its ability to augment cellular proliferation [8]. To investigate the effects that Smyd2 may have on cell proliferation, NIH3T3 cells were transfected with either Smyd2-Myc or Smyd3-Myc. Relative to control and consistent with previous findings [8], over-expression of Smyd3 markedly increased cell growth (Fig. 4). Conversely, the transfection of 3T3 cells with Smyd2 led to a decrease in their proliferation (Fig. 4), indicating a potential role for Smyd2 in the maintenance of cell-cycle progression.

## Discussion

Great strides have been made in the interpretation of covalent histone modifications regarding their role in transcriptional regulation. Histone lysine methylation has been found to affect the structure of chromatin thereby establishing complex patterns of gene expression [23]. In some cases, these patterns are clearly defined. For example, H3K4 methylation is most often associated with the establishment of euchromatin and the consequent activation of local gene expression [3]. Reciprocally, methylation at H3K9 is commonly involved with the formation of heterochromatin and the ensuing silencing of nearby gene transcription [3,4].

Initial data on the yeast HKMT, Set2, indicated that it functions in transcriptional repression by methylating H3K36 [24]. However, the HKMT activity of Set2 was later linked to the elongation phase of RNA polymerase II (RNAPII) [25,26]. Likewise, in a more contemporary study, an analysis of the distribution of H3K36 methylation in metazoans correlated this modification with actively transcribed genes [11]. Most recently, methylation of H3K36 by Set2 has been associated with the recruitment of a histone deacetylase complex, Rpd3 [13]. The overall role and implications of histone deacetylation within the coding regions of active genes is still unknown.

In mammalian epigenetics, NSD1 was one of the first HKMTs reported to act on H3K36 [27]. Whether NSD1 functions in the activation or repression of transcription has yet to be determined. A recent investigation reported that the human HYPB protein methylates H3K36 and that this enzymatic activity is required for the role of HYPB as a transcriptional activator [28].

Our findings introduce Smyd2 as an H3K36-specific HKMT that acts as a transcriptional repressor. Clearly, there are other transcriptional regulatory mechanisms at work in conjunction with the methylation of H3K36. It seems that the more we learn about where histone marks are localized and what proteins facilitate the process, the less we are certain about how such localization ultimately contributes to gene regulation. Although this complicates our ability to apply a broad interpretation of histone modifications, it provides a clear direction for the pursuit of a deeper fold in the "histone code."

### Smyd2 regulatory functions

Transcriptional assays demonstrated that Smyd2 can repress transcription from a luciferase reporter gene (Fig. 3B). A recent study in yeast demonstrated that methylation of H3K36 recruits a histone deacetylase complex, Rpd3 [13]. Concurrently, another group concluded that H3K36 methylation-induced recruitment of an Rpd3 complex resulted in the reversal of lysine acetylation related to the elongation phase of RNAPII, suggesting that it functioned to stem intragenic transcription initiation [14]. This is reminiscent of the mechanism by which the FACT complex functions. That is, as the elongation complex traverses a coding region, FACT facilitates both destabilization of the chromatin structure, to impart efficient and processive elongation, as well as reorganization of the chromatin to prevent intragenic initiation of transcription [29]. Whereas H3K36 methylation recruits the Rpd3 complex, it has been suggested that FACT recruitment may occur through its association with CHD1, which recognizes trimethylated H3K4 [30]. As the Rpd3 complex is known to contain Sin3 [13], it was particularly informative to find that Smyd2 also associates with Sin3. It will be of further interest to determine whether *in vivo *recruitment of Sin3 requires H3K36 methylation, the presence of Smyd2, or both.

Over-expression of Smyd2 in NIH3T3 cells significantly reduces cell growth. In a previous study, cell proliferation assays demonstrated that Smyd3 augmented cell growth when introduced into NIH3T3 cells [8]. It is well established that cell proliferation and differentiation are coordinated by synchronized patterns of gene transcription. In the case of Smyd3, enhancement of cell growth has been shown to be dependent upon the H3K4-specific HKMT activity of the Smyd3 protein [8]. It will be informative to determine whether the suppressive effect of Smyd2 on cell growth requires its function as an H3K36-specific HKMT. Such a determination, in tandem with identification of putative gene target specificity of Smyd2 will provide a broader mechanistic model of how the Smyd family may function.

Histone lysine methylation is more stable than other known post-translational modifications, persisting as long as several rounds of cell division [31-33]. This makes lysine methylation potentially valuable in diverse, long-lasting signaling networks, not only in the nucleus for histone and non-histone proteins, such as p53 and TAF10, but conceivably in the cytoplasm. The findings that Smyd1 and Smyd3 can localize in the cytoplasm [21,8] along with our observation that Smyd2 is also capable of cytosolic localization, lends credence to this idea. This argument is further strengthened by the finding that Smyd1 moves from the nucleus to the cytoplasm during myogenic differentiation [21]. Another SET domain-containing HKMT, Ezh2 and its partners Eed and Suz12, reside primarily in the cytoplasm of various mouse and human cells [34,35]. Within the nucleus, the Ezh2 complex catalyzes H3K27 methylation, whereas the cytosolic Ezh2 binds Vav1, a controller of Rho family GTPases, and Ezh2 is important for signaling events previously attributed to Vav1 [34-36]. There is no evidence that Ezh2 methylates Vav1, so the significance of lysine methylation in the cytoplasm remains unclear. However, we are currently testing the role of Smyd2-mediated lysine methylation in the formation of stable and potentially heritable cytosolic signaling complexes with Smyd2 interaction partners and we plan to track these complexes, once formed, within resting and dividing cells.

### The Smyd family

The Smyd HKMTs are set apart from other such chromatin modifying enzymes by the split nature of their SET domains. The SET domain of each Smyd protein is divided by a MYND domain (Figure 1A &1C), a zinc-finger motif that mediates protein-protein interactions. This domain is found in several transcriptional regulators shown to mediate distinct biological functions [37,38]. For example, the MYND domain of Smyd1 is essential for its interaction with the muscle-specific transcription factor, skNAC [21]. Additionally, ETO, a common target of chromosomal translocations in acute myeloid leukemia, directs transcriptional repression through an intact MYND domain [39]. Thus, the importance of the MYND domain in gene regulation has been well established and it may provide some insight into other mechanisms at work through Smyd2 that affect the overall outcome of its activity in transcriptional regulation. The complete function of Smyd2 *in vivo *is likely dependent upon other proteins and complexes, in addition to HDAC1 and Sin3A, with which it associates. We are currently screening several other candidate interaction partners whose nature may give further clues to mechanisms and pathways regulated by Smyd2.

Northern blot analysis revealed that Smyd2 and Smyd3 are expressed in a wide variety of tissues (Fig. 1B) whereas Smyd1 is more restricted in its tissue distribution [20]. Studies of ours and others on Smyds1-3 suggest that Smyd family members function through a common mechanism, specifically, lysine methylation. It is reasonable to assume that individual Smyd proteins associate with different transcription factors and other effector proteins that ultimately dictate specific gene regulation. No other Smyd family member is functionally redundant with Smyd1, since homozygous Smyd1-null mice are embryonic lethal at day E10.0 as a result of impaired cardiomyocyte differentiation [17]. The significant expression of Smyd2 in the in embryonic heart (Fig. 1B, E) suggests that as with Smyd1, Smyd2 may regulate cardiac development. The identification of the biological functions of Smyd family proteins will undoubtedly reveal new insights into the relationships between chromatin modifications and the development and differentiation of specific tissues.

## Conclusion

We conclude that Smyd2 dimethylates H3K36 and that HDAC1-mediated deacetylation of the coding regions of active genes is directly linked to this histone methyltransferase activity of Smyd2. We further propose that this role of Smyd2 in the regulation of gene expression ultimately restrains cell proliferation. As it is clear from this study, future research on Smyd proteins, with strong emphasis on the unique organismal context, will shed light onto the biological functions of Smyd family proteins, revealing new and fascinating insights into the relationships between chromatin modifications and the development and differentiation of tissues and organisms.

## Methods

### Computational analysis

The Smyd family members were identified from BLAST comparisons using the protein databases found at the National Center for Biotechnology Information (NCBI) web site . The ClustalW and BOXSHADE programs were used for alignments and shading of Smyd family proteins

### Cell culture

Cells lines were grown in DMEM, supplemented with 10% fetal bovine serum, 1 mM L-glutamine, 1% non-essential amino acids, penicillin, streptomycin, and fungizone (all from Life Technologies), at 37°C in a humidified atmosphere of 5% CO_2_. We used 293T, 10T1/2 and NIH3T3 cells, obtained from ATCC, in this study.

### Constructs

The SV40-luciferase reporter, containing five copies of the GAL4-UAS, was obtained from J. Milbrandt [25]. pRL-TK was purchased from Promega. The GAL4-mammalian expression construct, GAL4-DBD (DNA-binding domain), has been described previously [17]. The Smyd2 and Smyd3 full-length EST clones were obtained from Invitrogen. The GAL4-Smyd2 mammalian expression vector was constructed by PCR amplification (5' ATG CGC GCC GAG GCC CGC; 3' TCA GTG GCT CTC AAT CTC CTG) and restriction digestion (Not I; Xba I) followed by subcloning into the GAL4-DBD plasmid [17]. The Myc-Y240F and Myc-Y239F point mutations were generated within the SET domain of Myc-Smyd2 (5' CGA GGT GTT CAC CAG CTT CAT CGA CCT GCT ATA TCC; 3' GGA TAT AGC AGG TCG ATG AAG CTG GTG AAC ACC TCG) and Myc-Smyd3 (5' GGA GCT CAC CAT CTG CTT CCT GGA CAT GCT GAT GAC C; 3' GGT CAT CAG CAT GTC CAG GAA GCA GAT GGT GAG CTC C), respectively, using the GeneEditor *in vitro *Site-Directed Mutagenesis System (Promega) according to the instructions of the manufacturer. The FLAG-tagged HDAC1-F mammalian expression plasmid was a kind gift from S.L. Schreiber [40]. GST- fusion proteins were prepared in *E. coli *and purified using glutathione agarose beads (Sigma-Aldrich). GST- cleavage was accomplished using PreScission Protease (Amersham bioscience); purity of the products was verified using silver staining of SDS-PAGE gels. The FLAG-tagged MBD3-F and MTA2-F expression plasmids were supplied by R.M. Evans [41]. The sequences of all constructs were confirmed by DNA sequencing.

### Transient transfections and luciferase assays

All transfections were performed using FuGENE6 reagent (Roche), according to the instructions of the manufacturer. For immunoprecipitation experiments, 293T cells were plated at a density of 2 × 10^6 ^cells per 100 mm plate 24 hours prior to transfection. 8 μg of total DNA plus 24 μl of FuGENE6 reagent was used per 100 mm plate. Cells were harvested 48 hours after transfection. For luciferase reporter assays, 10T1/2 cells were seeded at a density of 2 × 10^5 ^cells per 6-well plate 24 hours prior to transfection. 3.5 μg of total DNA plus 10.5 μl of FuGENE6 reagent was used per well. Luciferase assays were performed using the Dual-Luciferase Reporter Assay System kit (Promega) according to the manufacturer's directions. Samples were read using a Dynex microtiter luminometer.

### Antibodies

The anti-Myc mouse monoclonal (9E10) and monoclonal anti-FLAG (M2) antibodies were purchased from Sigma-Aldrich. The anti-GAL4 mouse monoclonal (RK5C1) and the anti-Sin3A rabbit polyclonal (K-20) antibodies were purchased from Santa Cruz Biotechnology. Antibodies for determining the specificity of HKMT activity are detailed below. Peroxidase-conjugated whole IgG secondary antibodies were purchased from Jackson ImmunoResearch Laboratories. Western blotting and immunoprecipitation experiments were conducted as described previously [21].

### Multiple tissue northern analysis

Northern blotting was performed using ULTRAhyb Hybridization Buffer (Ambion) according to the instructions of the manufacturer. The probes for full-length Smyd2 and Smyd3 were generated by restriction digest excision (Not I; Xba I) from their respective GAL4 mammalian expression constructs and the Strip-EZ DNA Probe Synthesis Kit (Ambion) as described in the manual. The probe for full length Smyd1 was generated by restriction digest excision (EcoRI) from the pBK-CMV-Smyd1B expression construct described previously [21]. The multiple tissue Northern blot was purchased from Ambion (FirstChoice Northern Blot Mouse Blot I). Blots were detected using a phosphoimager.

### *In situ *hybridization

The DNA probe for full-length Smyd2 was generated by restriction digest excision (Not I; Xba I) from its above described GAL4 mammalian expression construct. Probe synthesis, hybridization, and autoradiography were performed as described in Lu et al. [42] with slight modifications. Briefly, embryos were obtained at day 13.5 post coitus and fixed overnight in 4% paraformaldehyde. Hybridization of tissue sections with sense and antisense DNA probes was performed overnight at 55°C, 7.5 × 10^5 ^cpm per slide. Unhybridized probe was removed through stringent washing and slides were coated with K.5 nuclear emulsion (Ilford, UK) followed by exposure at 4°C for 21 days. Once developed, slides were counterstained with hematoxylin and observed by bright and dark field optics.

### Cellular localization by fluorescence imaging

293T cells were transfected with 1 μg of plasmids, encoding myc tagged Smyd2, using Fugene 6 (Roche), according to the manufacturer's instructions. 48 hr post-transfection, cells were fixed (4% paraformaldehyde in PBS for 10 min), washed, and permeabilized (0.5% Triton X-100 in PBS for 15 min). Monoclonal mouse anti-myc antibody was incubated 45 min at room temperature. After washing, goat anti-mouse Alexa 488 (Molecular Probes) was diluted 1:400 in 2% BSA and incubated 15 min at room temperature in the dark. Nuclei were counterstained with DAPI. Slides were washed twice for 45 min and mounted with VectaStain. Cells were analyzed by sequential confocal laser scanning microscopy (Leica SP2 AOBS).

### Cell proliferation assay

NIH3T3 cells were transfected with 6 μg of Smyd2-Myc, Smyd3-Myc or pcDNA3 alone. The effect of the over-expression of each protein on cell growth was observed over a 6-day period and evaluated by cell counting using trypan-blue staining.

### In vitro histone methyltransferase assay

293T cells were transfected with plasmids expressing Myc-tagged wildtype Smyd2 (Smyd2-Myc), wildtype Smyd3 (Smyd3-Myc), mutant Smyd2 (Smyd2 (Y240F)-Myc), or mutant Smyd3 (Smyd3 (Y239F)-Myc) and the over-expressed, tagged proteins were purified by immunoprecipitation with an anti-Myc antibody. The histone methyltransferase assay was performed as described in Hammamoto et al. [8]. Briefly, the Smyd proteins were incubated with 1 μg of mixed histones from calf thymus (Sigma) or recombinant H3 (Upstate). In addition, 2 μCi S-adenosyl-L – [methyl-^3^H ] methionine (SAM; Amersham Biosciences) was included as a methyl donor. All reactions were carried out in 40 μl HKMT reaction buffer (10 mM dithiothreitol, 100 mM NaCl, and 50 mM Tris-HCl at pH 8.8) at 30°C for 3 hours. An 18% SDS-PAGE gel was used to resolve the samples and fluorography was used visualize positive methylation. Substrate loading was visualized by Coomassie blue staining.

To determine the specificity of Smyd2 activity, immunoprecipitated Smyd proteins were incubated with recombinant H3 and 20 μM unlabelled SAM (Sigma) in 40 μl HKMT reaction buffer at 30°C for 1 hour. Western blot analysis was conducted using antibodies against dimethylated H3K4, trimethylated H3K4, dimethyl H3K9, trimethyl H3K9, trimethyl H3K27, dimethyl H3K36, or dimethyl H3K79 (all from Upstate, Charlottesville, VA, USA).

## Competing interests

The author(s) declare that they have no competing interests.

## Authors' contributions

All authors participated in the design of experiments. M.A.B. was responsible for northern analyses, cell localization studies, histone methyltransferase assays, and cell proliferation assays. M.A.B. and R.J.S. performed all western blots and prepared all of the novel constructs. R.J.S. was responsible for protein interaction data, luciferase assays, protein alignments, and critical examination of the manuscript. P.D.G. and P.W.T. coordinated and acquired funding for the study. P.W.T. revised the manuscript. All authors read and approved the final manuscript.
